# Dose Volume and Liver Function Test Relationship following Radiotheraphy for Right Breast Cancer: A Multicenter Study

**DOI:** 10.3390/curroncol30100632

**Published:** 2023-09-26

**Authors:** Zeliha Güzelöz, Oğuzhan Ayrancıoğlu, Nesrin Aktürk, Merve Güneş, Zümre Arıcan Alıcıkuş

**Affiliations:** 1 Department of Radiation Oncology, Health Science University Tepecik Training and Research Hospital, İzmir 35100, Türkiye; 2 Department of Radiation Oncology, İzmir Tınaztepe University Galen Hospital, İzmir 35001, Türkiye; oguzhan.ayrancioglu@tinaztepe.com (O.A.); merve.gunes@tinaztepe.com (M.G.); zumre.arican@tinaztepe.edu.tr (Z.A.A.); 3 Department of Radiation Oncology, Katip Çelebi University Atatürk Training and Research Hospital, İzmir 35150, Türkiye; nesrin.akturk@saglik.gov.tr

**Keywords:** right breast, radiotherapy, liver function tests, dose–volume, toxicity

## Abstract

Objective: The liver is a critical organ at risk during right breast radiotherapy (RT). Liver function tests (LFTs) such as alanine aminotransferase (ALT), aspartate aminotransferase (AST), and gamma-glutamyl transferase (GGT) serve as biochemical markers for hepatobiliary damage. In this multicenter cross-sectional study, the effects of liver dose–volume on changes in LFTs pre- and post-RT in patients treated for right breast cancer were evaluated. Materials and Methods: Between January 2019 and November 2022, data from 100 patients who underwent adjuvant right breast RT across three centers were retrospectively assessed. Target volumes and normal structures were contoured per the RTOG atlas. Patients were treated with a total dose of 50 Gy in 25 fractions to the CTV, followed by a boost to the tumor bed where indicated. The percentage change in LFT values in the first two weeks post-RT was calculated. Statistics were analyzed with SPSS version 22 software, with significance set at *p* < 0.05. Statistical correlation between liver doses (in cGy) and the volume receiving specific doses (Vx in cc) on the change in LFTs were analyzed using Kolmogorov–Smirnov, Mann–Whitney U test. Results: The median age among the 100 patients was 56 (range: 29–79). Breast-conserving surgery was performed on 75% of the patients. The most common T and N stages were T1 (53%) and N0 (53%), respectively. None of the patients had distant metastasis or simultaneous systemic treatment with RT. A total of 67% of the treatments utilized the IMRT technique and 33% VMAT. The median CTV volume was 802 cc (range: 214–2724 cc). A median boost dose of 10 Gy (range: 10–16 Gy) was applied to 28% of the patients with electrons and 51% with IMRT/VMAT. The median liver volume was 1423 cc (range: 825–2312 cc). Statistical analyses were conducted on a subset of 57 patients for whom all three LFT values were available both pre- and post-RT. In this group, the median values for AST, ALT, and GGT increased up to 15% post-RT compared to pre-RT, and a median liver D_mean_ below 208 cGy was found significant. While many factors can influence LFT values, during RT planning, attention to liver doses and subsequent regular LFT checks are crucial. Conclusion: Due to factors such as anatomical positioning, planning technique, and breast posture, the liver can receive varying doses during right breast irradiation. Protecting patients from liver toxicity secondary to RT is valuable, especially in breast cancer patients with a long-life expectancy. Our study found that, even in the absence of any systemic treatment or risk factors, there was an average increase of nearly 15% in enzymes, indicating acute liver damage post-RT compared with pre-RT. Attention to liver doses during RT planning and regular follow-up with LFTs is essential.

## 1. Introduction

Breast cancer is the most frequently diagnosed cancer among women worldwide [1]. Adjuvant radiation therapy (RT) plays a pivotal role in the treatment of breast cancer [2,3]. There has been an observed increase in locoregional control in patients undergoing breast-conserving surgery with adjuvant RT and selected patients receiving mastectomy [4,5]. Following breast-conserving surgery, RT to the preserved breast halves the local recurrence rate and lowers breast cancer mortality by approximately one-sixth [5].

The success achieved in locoregional control with RT has also reflected positively in survival rates [4,5,6]. Variations in local treatments that have a significant impact on local recurrence rates would, under the assumption of no other causes of death, prevent approximately one breast cancer-related death within the next 15 years for every four avoided instances of local recurrence, consequently leading to a decrease in overall mortality over the course of 15 years [5]. Nowadays, due to the diffusion of breast cancer screening programs and advancements in imaging technology, breast cancer diagnoses are being made at younger ages [7,8]. This means that younger-patient populations need to be followed for many years. Advances in both RT and systemic treatments have improved the prognosis of these patients, emphasizing the importance of the quality of life and preservation of normal tissue. Particularly with the increasing young patient population, there has been a growing emphasis on the need for better protection of normal tissues during RT. Protecting these long-surviving patients from acute side effects is just as crucial as minimizing secondary cancer risks in the long term.

For many years, numerous studies have been conducted on radiation-induced liver disease (RILD). Especially in patients undergoing abdominal RT, the liver stands as one of the priority normal tissues to be protected [9]. In right breast RT practices, due to anatomical proximity, the liver is one of the normal tissues at risk. However, the etiology of RILD is multi-factorial, with a central role of veno-occlusive processes and, although as low dose exposure may as well exert some effects, no specific liver dose constraints have been defined in the setting of adjuvant breast irradiation [10].

The liver, being a metabolic organ with vital functions, has liver function tests (LFTs) such as alanine aminotransferase (ALT), aspartate aminotransferase (AST), and gamma-glutamyl transferase (GGT), which are biochemical indicators of hepatobiliary damage for various reasons. The normal ranges for ALT, AST, and GGT are 0–45 IU/L, 0–35 IU/L, and 0–45 IU/L, respectively [10]. In the literature, there are limited studies examining long- and short-term changes in LFTs post-RT [11,12,13,14]. Grade 3–4 hepatotoxicity was not identified in these few studies. However, a correlation was found between irradiated liver volume and ALT and ALP tests [11]. A significant increase was detected in IL-6 level [12]. An increase in median AST and ALT values was observed after radiotherapy [13].

In this multicentric cross-sectional study, the aim was to evaluate the impact of liver dose–volume on changes in LFT values before and after RT in patients treated for right breast cancer.

## 2. Patients and Methods

### 2.1. Patient Selection

In this study, data from 100 female patients aged 18 and over who underwent RT to the right breast or right chest wall following breast-conserving surgery or mastectomy between January 2019 and November 2022 in three centers with identical RT protocols were retrospectively evaluated. These patients had a diagnosis of invasive breast carcinoma without distant organ metastasis and had pre-radiation therapy (preRT) and post-radiation therapy (postRT) liver function test values (AST, ALT, GGT). Staging was performed according to the American Joint Committee on Cancer tumors, lymph nodes, and distant metastases TNM staging system (8th ed., 2017). Patients diagnosed with stage IV or in situ carcinoma, those who received neoadjuvant chemotherapy, those undergoing concurrent systemic treatment, or those with chronic liver or biliary tract disease were excluded from this study. The study protocol was approved by the national ethics committee (Health Science University Tepecik Training and Research Hospital Ethics Committee approval number: 2023/07-05).

### 2.2. Radiation Therapy

#### 2.2.1. Simulation

All patients were planned in a supine position using a breast board with arm support. Tomographic slices were acquired at intervals of 3 mm. In the acquired topographies, the entire liver was included in the imaging field.

#### 2.2.2. Contouring of Target Volumes

Target volumes and at-risk normal tissues were contoured on the tomographic slices taken at a 3 mm slice thickness according to the Radiation Therapy Oncology Group (RTOG)/European Organization for Research and Treatment of Cancer (EORTC) guidelines [15]. For patients who underwent breast-conserving surgery, the entire right mammary glandular tissue and skin were determined as breast CTV (clinical target volume). Lumpectomy cavities and seromas were included in the CTV. For patients who underwent mastectomy, the chest wall including the incision scar and skin was contoured. PTV (planning target volume) was obtained by giving a five mm margin to CTV.

#### 2.2.3. Contouring of the Liver

The liver was contoured based on the RTOG upper abdomen normal tissue contouring guidelines [16]. The entire liver in the slice area was contoured in the abdomen window level range. The gallbladder was excluded. The portal vein, branches of the portal vein, and other vessels were included within the liver (except inferior vena cava) contour according to the guidelines [17].

#### 2.2.4. Radiotherapy Prescription and Planning

Patients received a total of 50 Gy RT over 25 fractions of CTV using FIF (field in field)/IMRT (intensity-modulated radiotherapy)/VMAT (volumetric arc therapy) techniques. Where necessary, an additional dose (boost) was given to the tumor bed using either electron or photon energy. The energy of 6–10 MVX was utilized. It was aimed to keep the volume of the right lung receiving 20 Gy below 30%.

#### 2.2.5. Liver Dose–Volume

Assessment from the dose–volume histogram, values for the D_max_ (maximum dose, D_min_ (minimum dose), D_mean_ (mean dose), and (V_x_) the volume of the liver (cc) receiving a certain dose (x) were (V_5_, V_10_, V_20_, V_30_, V_40_, and V_50_) recorded. According to normal tissue dose limitations, the mean dose to the liver was aimed to be below 30–32 Gy [18].

### 2.3. Laboratory Tests

ALT, AST, and GGT blood values from two weeks before the initial fraction of RT (preRT) and two weeks after the last fraction of RT (postRT) were obtained from hospital and national medical record systems.

### 2.4. Statistics

The percentage difference (Δ%) for each of the three parameters between preRT and postRT was calculated using the formula Δ% = (postRT − preRT)/preRT × 100. Based on this formula, a positive percentage difference indicated an increase in LFTs after RT, while a negative value indicated a decrease post-RT. The effects of liver doses (cGy) and volumes (Vx) (cc) on Δ% were evaluated. Statistics were analyzed with SPSS© 22 software (Statistical Package for the Social Sciences), with significance set at *p* < 0.05. Statistical correlation between liver doses (in cGy) and the volume receiving specific doses (Vx in cc) on the change in LFTs were analyzed using Kolmogorov–Smirnov, Mann–Whitney U test.

## 3. Results

The demographic and treatment data of the patients can be seen in Table 1.

After radiotherapy, it was observed that AST values were above the normal range in 12 patients (ranging from 45 to 1107 IU/L), ALT values in 12 patients (ranging from 35 to 365 IU/L), and GGT values in 12 patients (ranging from 49 to 414 IU/L).

No patient received systemic therapy or tamoksifen concurrent with RT. The median liver volume was 1423 cc, with a range of 825–2312 cc. The median D_min_ was 3.4 cGy (range: 0–206.1 cGy), the median D_max_ was 4814 cGy (range: 110–206.1 cGy), and the median D_mean_ was 203 cGy (range: 15–1497 cGy). The observed dose–volume values were as follows: Median V_50_ was 0 cc (range: 0–68), V_40_ was 0.76 cc (range: 0–87.2), V_30_ was 2.14 cc (range: 0–180.7), V_20_ was 6 cc (range: 0–387.7), V_10_ was 11.7 cc (range: 0–949.1), and V_5_ was 21.2 cc (range: 0–1352).

**Figure 1:**
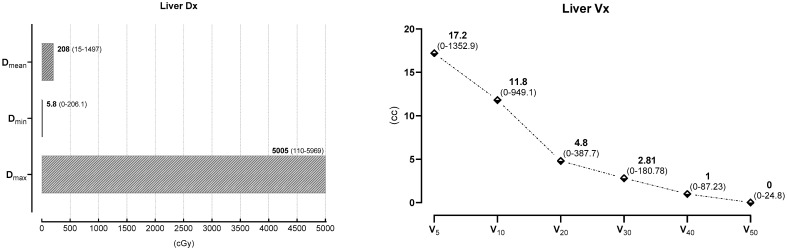
Liver Dosimetric and V_5–50_ Values.

For the statistical analyses, 57 patients were included, for whom all three LFTs were completely obtained in both the pre- and post-RT periods. In this patient group, the median CTV volume was 806 cc (range: 214–2519 cc) and the median liver volume was 1457 cc (range: 825–2218 cc). The D_max_, D_min_, and D_mean_ dose values are presented in Table 2 and Figure 1, while the liver V_5–50_ dose values are shown in Table 3 and Figure 1.

**Figure 2:**
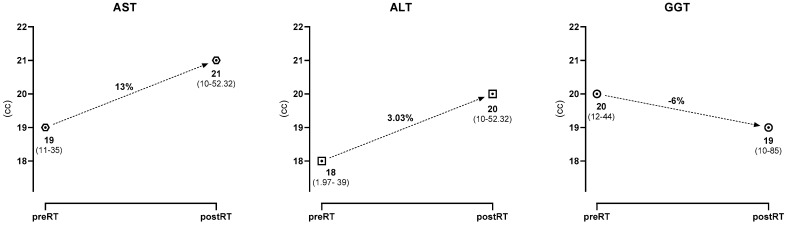
Median and percentage change in liver function test (LFT) values of 57 patients.

The median values and percentage changes in ALT, AST, and GGT tests prior to and following RT are provided in Table 4 and Figure 2.

When examining the effect of liver dose–volume on the percentage change between preRT and postRT in LFT, a statistically significant adverse effect was observed with higher liver D_mean_ (*p* = 0.03) values solely for ALT and for AST with both liver D_min_ (*p* = 0.007) and D_mean_ (*p* = 0.023) values. For GGT, all liver dose–volume values, namely D_min_ (*p* = 0.014), D_max_ (*p* = 0.023), D_mean_ (*p* = 0.006), V_50_ (*p* = 0.009), V_40_ (*p* = 0.03), V_30_ (*p* = 0.03), V_20_ (*p* = 0.001), V_10_ (*p* = 0.02), and V_5_ (*p* = 0.008), were found to be statistically significant. However, the RT technique, CTV volume, the addition of boost, and its technique did not demonstrate a statistically significant effect. The statistically significant values, effect of liver dose–volume and the percentage change are presented in Table 5.

## 4. Discussion

Radiation in the early phase results in DNA damage, oxidative stress, and an accumulation of free oxygen radicals in the environment, leading to acute inflammation and hepatocellular apoptosis [19]. This scenario creates vascular damage that subsequently results in an increased synthesis of collagen, negatively impacting growth factors, TNF-alpha, TNF-beta, and other elements involved in liver damage regulation and repair [20]. Clinically, this situation is recognized as radiation-induced liver disease (RILD).

Classic RILD is observed between 2 weeks and 4 months post-radiation in patients who have received 30–35 Gy through conventional fractionation of the liver [21]. It arises due to veno-occlusion associated with fibrosis secondary to RT. Its presentation involves an ALP level increased by ≥2 times. With advancements in radiation technology, such as image-guided RT techniques, VMAT plans, IMRT plans, and stereotaxic body radiotherapy, classic RILD has become less common. Instead, non-classic RILD is more frequently observed. In this scenario, even with a lower radiation dose, there can be a rise in LFTs, possibly due to diminished liver regeneration capacity, which may be associated with conditions like cirrhosis or hepatitis [10,21]. In such cases, AST and ALT levels may elevate to ≥5 times [13].

Anatomically, the liver is near the radiation treatment area during breast or chest wall irradiations, particularly on the right side, making it an at-risk organ. Current dose restrictions used in planning RT for right breast cancer recommend a D_mean_ value of 28–32 for the liver [18]. This dose carries a 5% risk of developing RILD [22]. However, when considering the anatomy and the conventional dose of 50 Gy given to the entire breast, this prescribed dose for the liver seems excessively high and is not reflective of reality. Considering the ALARA principle “As Low As Reasonably Achievable”, these theoretically appropriate dose limitations pose different challenges in clinical practice. This principle aims at minimizing the risks associated with radiation exposure, thus striving to keep radiation doses in diagnostic and therapeutic processes as low as reasonably achievable. Within the framework of this principle, the use of radiation at necessary therapeutic doses aims to minimize acute and chronic side effects that may occur following RT. Consequently, the objective is to reduce the long-term risk of secondary cancer development attributed to RT.

In studies assessing liver doses in patients diagnosed with breast cancer and treated with right breast irradiation, the mean liver dose was found to be between 1.94 and 4.34 Gy [11,13,14]. The maximum liver dose averages at 26.9 Gy and in some cases reaches as high as 51.7 Gy [14]. There are limited studies in the literature that focus on liver function alterations due to the dose received by the liver during right breast irradiation. You can see these studies in Table 6.

In our study, unlike in the literature, early changes in LFTs were calculated as a percentage change using a mathematical formula, and the relationship between this value and dose–volume values was evaluated. It was determined that, as the mean dose received by the liver increases, there is a significant increase in ALT and AST values (*p* = 0.03, *p* = 0.023 respectively). Furthermore, it has been shown that the higher the minimum dose the liver receives, the greater the increase in AST value (*p* = 0.007). Therefore, keeping the mean and minimum dose received by the liver as low as possible is seen as one of the essential parameters to avoid LFT increase. A statistically significant decrease in percentage change and GGT values was observed after RT. This could be attributed to the GGT levels not being negatively affected during the acute phase of RT.

In the current study, no significant relationship between percentage difference (Δ%) and a certain volume dose (VxGy) was not detected. In the literature, it is recommended that the liver receives a dose below 30 Gy (V_30_ < 100%). It is argued that a dose above 30 Gy is an indicator of RDIL [17,23,24,25]. In our study, the median V_30_ value was found to be 2.81 cc, which corresponds to approximately 2% of the median value. We think that, since such a low value was found, there was no clinical change and no relationship was detected with DVH. One of the key points should be the actual clinical impact of low-dose exposure to the liver. The liver is well-know for its ability to regenerate after multiple kinds of damage. Several previous experiences demonstrated that, although RT could result in increased LFT, it did not meet the criteria for RILD [13] and delayed hepatotoxicity was negligible, questioning the definition of liver as an OAR [14]. In a study by Park et al. evaluating LFTs in patients diagnosed with breast cancer undergoing RT, it was reported that 53.6% of the patients had a V_30_ value of 0 and the maximum V_30_ value was 2.6%, and RILD was not observed in any patients. Based on this, it has been suggested to use a liver D_mean_ ≤ 3–4 value as the liver normal tissue dose limitation for right breast irradiation and can be considered as a cut-off value [13]. The similar low doses found in our study and absence of changes in LFTs support this thesis.

Survival rates have increased in patients diagnosed with breast cancer due to advancements in RT techniques and progress in systemic treatments. It is possible to observe the long-term stochastic effects of radiation, which are independent of dose, in the patient group monitored with a breast cancer diagnosis. Therefore, the incidence of secondary cancers after breast cancer irradiation during follow-up is higher than that for other types of cancer [26,27]. Even if the results do not manifest clinically as an increase in LFTs, considering the long-term effects of the received radiation, normal tissues should be exposed to the lowest possible radiation dose, as discussed in accordance with the ALARA principle [28]. Radiation-induced cancer is classically defined as a stochastic process, although recent studies developed more complex models; therefore, there is no threshold point and even low doses may increase second neoplasms risk. This phenomenon is relevant especially for long-term survivors and has been extensively investigated for lymphoma and breast cancer patients, mostly focusing on second lung, breast and thyroid malignancies [29,30,31]. Nonetheless, some studies defined the risk of secondary liver cancer after breast irradiation, with conflicting results: while in some models, the lifetime attributable risk (LAR) for liver cancer induction after breast radiotherapy was extremely low [32], in other experiences high LAR estimates were obtained for liver in case of right-sided targets [33].

Currently, the deep inspiration breath hold (DIBH) is employed as standard in left breast and chest wall irradiation. This technique is used in left breast cancer RT to ensure that cardiac tissues and coronary arteries receive a lower dose [34,35,36]. The DIBH technique has not yet become standard for right breast or chest wall RT. There are fewer studies on the benefits of the DIBH technique in right breast irradiation. While there are studies that determined that it reduces the dose to the heart, lungs, and liver dosimetrically [37], there are also studies that argue it is effective in reducing liver doses only in cases with hepatomegaly while reducing doses to the heart and LAD (left anterior descending artery) [38]. In the study of Loap and colleagues, although there was no significant change in cardiac structures and the right lung in right breast irradiations using DIBH compared to the free breath technique with VMAT, a significant reduction was observed in the mean liver dose (from 2.54 to 0.87 Gy *p* = 0.001). Therefore, it has been emphasized that, instead of routine use, it should be used in selected patients [39].

Due to its retrospective design, our study inherently possesses some limitations. Despite the availability of 100 patients that met the study criteria, statistical analysis was performed on the 57 patients with data for all three liver function test parameters. None of the patients included in the study received concurrent chemotherapy and tamoxifen alongside RT. Some patients received neoadjuvant or adjuvant chemotherapy. As per our protocol, RT begins approximately 3–4 weeks after chemotherapy. The reason for conducting LFTs just before RT is to assess the reduction in potential toxicity that could occur due to chemotherapy during this period. Furthermore, since the primary focus of our study is on the changes occurring in the acute phase before and after radiation therapy, the effect of chemotherapy has not been separately evaluated. On the other hand, according to the literature, it is known that hormonal therapies used in the post-menopausal period (like letrozole and exemestane) do not have an effect that will reflect on the clinic and tests [40,41]. Although there is a viewpoint that minimal changes in LFTs may not have clinical implications, it is essential to remember that slight elevations in AST, ALT, and GGT due to scattered radiation may indicate potential risks concerning non-RILD and secondary cancers in the long run.

There is a dearth of research in the literature that examines early changes in LFTs after right breast irradiation. We aimed to address this gap. The multicentric design of our study, its evaluation using modern RT techniques, the detailed examination of DVH parameters, and the articulation of LFT changes through a mathematical formula constitute this study’s strengths.

## 5. Conclusions

In conclusion, liver damage can manifest as a spectrum ranging from subtle laboratory abnormalities to severe liver insufficiency. Due to factors such as anatomical positioning, planning technique, and breast posture during right breast irradiation, the liver can receive variable doses. For breast cancer patients with a longer survival expectancy, safeguarding them from potential liver toxicity secondary to RT is of paramount importance. Our findings indicate that, in patients who did not undergo any systemic treatment or had no risk factors, there was an average increase of nearly 15% in enzymes, indicative of acute liver damage post-RT compared with pre-RT. It was deemed significant to maintain liver D_mean_ under 208 cGy. Given the myriad of factors influencing LFT values, our study underscores the necessity for meticulous attention to liver doses during RT planning. We advocate for maintaining the mean dose below 208 cGy and emphasize the importance of regular LFT monitoring during follow-up.

## Figures and Tables

**Table 1:** Demographics and treatment data of patients.

**Median Age**	56 (29–79)
Median CTV volume	802 (214–2724) cc
Surgery modality	
breast conserving	75%
mastectomy	25%
**T Stage**	
T1	53%
T2	39%
T3	-
T4	-
Tx	8%
**N Stage**	
N0	53%
N1	25%
N2	-
N3	-
Nx	22%
**RT technics**	
FIF/IMRT	67%
VMAT	33%
**Deep inspiration breath hold**	25%
**RT boost dose (median)**	10 (10–16) Gy
**RT boost**	
Electron	28%
IMRT	31%
VMAT	20%
Patient not received boost	21%

CTV: Clinical target volume; RT: Radiotherapy; FIF: Field in field; IMRT: Intensity-modulated radiotherapy; VMAT: Volumetric arc therapy.

**Table 2:** Liver Dosimetric Values (cc) of 57 patients.

Liver Dx	D_max_ (cGy)	D_min_ (cGy)	D_mean_ (cGy)
Dose (median)	5005 (110–5969)	5.8 (0–206.1)	208 (15–1497)

D_max_: Maximum dose; D_min_ Minimum dose; D_mean_: Mean dose; cGy centi Gray.

**Table 3:** Liver V_5–50_ Values (cc) of 57 patients.

Liver V_x_	V_5_	V_10_	V_20_	V_30_	V_40_	V_50_
cc (median)	17.2	11.8	4.8	2.81	1	0
	(0–1352.9)	(0–949.1)	(0–387.7)	(0–180.78)	(0–87.23)	(0–24.8)

V_5/10/20/30/40/50_ liver volume receiving 5/10/20/30/40/50 Gy.

**Table 4:** Median and percentage change in liver function test (LFT) values of 57 patients.

Liver Test	Median (U/L)	Median Percentage Change (%)
**AST**		
preRT	19 (11–35)	13% (−120 to 54.5)
postRT	21 (10–52.32)	
**ALT**		
preRT	18 (1.97–39)	3.03% (−292 to 46.1)
postRT	20 (8- 55)	
**GGT**		
preRT	20 (12–44)	−6% (−93.18 to 42.86)
postRT	19 (10–85)	

ALT: Alanine aminotransferase; AST: Aspartate aminotransferase; GGT: Gamma-glutamyl transferase; preRT: pre-radiation therapy; postRT: post-radiation therapy.

**Table 5:** Significant values of dose–volume and percentage chance on LFT.

Liver Test	Dose–Volume Parameters	*p* Value
**ALT**	D_mean_	0.03
**AST**	D_mean_	0.023
	D_min_	0.007
**GGT**	D_mean_	0.006
	D_min_	0.014
	D_max_	0.023
	V_50_	0.009
	V_40_	0.03
	V_30_	0.03
	V_20_	0.01
	V_5_	0.02

ALT: Alanine aminotransferase; AST: Aspartate aminotransferase; GGT: Gamma-glutamyl transferase; D_max_: Maximum dose; D_min_: Minimum dose; D_mean_: Mean dose; V_5/10/20/30/40/50_ Liver volume receiving 5/10/20/30/40/50 Gy.

**Table 6:** Studies examining LFT changes following right breast irradiation.

	The Number of Patients/RT Dose/Timing of Blood Test	Liver Dose	Hepatic Blood Test Results
Lauffer et al. [11]	34 right side 42.5 Gy/16 fr or 50 Gy/25 fr ±16 fr boosts Before and last week of RT	MLV: 1270.2 cc (918.5–2233.2) MLD: 1.94 Gy (0.2–9)	Correlation between irradiated liver volume and ALT (*p* = 0.05) and ALP (*p* = 0.006)
Courtier et al. [12]	52 right side, 100 left side 40 Gy/15 fr Before and during 4 weeks after RT	Mean V_10:_ 226 cm^3^ (19%) Mean V_50:_ 92 cm^3^ (8%) Mean V_90:_ 62 cm^3^ (5%)	V_10_ and IL-6 (*p* = 0.001)
Park et al. [13]	47 right side, 78 left side 42.56–50 Gy/16–25 fr ± 10–14 Gy boost 1 week before vs. 6 months after	D_mean_right breast_ 434.1 cGy D_mean_left breast_260.6 cGy V_10_ 3% V_20_ 1% V_30_ 0%	AST_median_: 23.2 ± 5.3 vs. 29.6 ± 14.6 ALT_median_: 20.2 ± 7.7 vs. 25.6 ± 20.0
Quintin et al. [14]	27 right side or bilateral, 29 left side Median follow-up 5.4 years	D_mean_ 2.8 Gy (0.3–16.6) D_max_ 26.9Gy (0.7–51.7)	no grade 3 hepatotoxicity Three patients (6%) with grade 2 delayed hepatotoxicity

RT: Radiotherapy; Gy: Gray; ALT: Alanine aminotransferase; AST:Aspartate aminotransferase; MLV: Mean lung volume; MLD: Mean lung dose; D_max_: Maximum dose; D_mean_: Mean dose; V_10/20/30/50/90_ volume of liver irradiated 10/20/30/50/90% of prescription dose.

## Data Availability

The data presented in this study are available on the request from the corresponding author.
